# Estimation of the National Surgical Needs in India by Enumerating the Surgical Procedures in an Urban Community Under Universal Health Coverage

**DOI:** 10.1007/s00268-020-05794-7

**Published:** 2020-09-24

**Authors:** Prashant Bhandarkar, Anita Gadgil, Priti Patil, Monali Mohan, Nobhojit Roy

**Affiliations:** 1 Department of Statistics, BARC Hospital, ‘WHO Collaboration Centre for Research in Surgical Care Delivery in LMICs’, Mumbai, India; 2 Department of Surgery, BARC Hospital, ‘WHO Collaboration Centre for Research in Surgical Care Delivery in LMICs’, Mumbai, India; 3 Consultant, ‘WHO Collaboration Centre for Research in Surgical Care Delivery in LMICs’, Mumbai, India; 4 Department of Global Public Health, Karolinska Institute, 17177 Stockholm, Sweden; 5 WHO Collaboration Centre for Research in Surgical Care Delivery in LMICs, Mumbai, India

## Abstract

**Background:**

11% of the global burden of disease requires surgical care or anaesthesia management or both. Some studies have estimated this burden to be as high as 30%. The Lancet Commission for Global Surgery (LCoGS) estimated that 5000 surgeries are required to meet the surgical burden of disease for 100,000 people in LMICs. Studies from LMICs, estimating surgical burden based on enumeration of surgeries, are sparse.

**Method:**

We performed this study in an urban population availing employees’ heath scheme in Mumbai, India. Surgical procedures performed in 2017 and 2018, under this free and equitable health scheme, were enumerated. We estimated the surgical needs for national population, based on age and sex distribution of surgeries and age standardization from our cohort.

**Result:**

A total of 4642 surgeries were performed per year for a population of 88,273. Cataract (22.8%), Caesareans (3.8%), surgeries for fractures (3.27%) and hernia (2.86%) were the commonest surgeries. 44.2% of surgeries belonged to the essential surgeries. We estimated 3646 surgeries would be required per 100,000 Indian population per year. One-third of these surgeries would be needed for the age group 30–49 years, in the Indian population.

**Conclusion:**

A total of 3646 surgeries were estimated annually to meet the surgical needs of Indian population as compared to the global estimate of 5000 surgeries per 100,000 people. Caesarean section, cataract, surgeries for fractures and hernia are the major contributors to the surgical needs. More enumeration-based studies are needed for better estimates from rural as well as other urban areas.

**Electronic supplementary material:**

The online version of this article (10.1007/s00268-020-05794-7) contains supplementary material, which is available to authorized users.

## Introduction

11% of the global burden of disease requires surgical or anaesthesia care or both [1, 2]. The Lancet Commission for Global Surgery (LCoGS) estimated 5000 surgeries are required to meet the surgical burden of diseases of 100,000 people in low- and middle-income countries (LMICs). The reported rates of surgery vary from 295 in LMICs to 23,000 in high-income countries (HIC) per 100,000 population. This variation is explained by unavailability of surgeons, anaesthetists, obstetricians (SAOs), lack of access to healthcare facilities [3] and underreporting of the surgeries that are performed. Addressing and enumerating surgical needs of the population, especially in low-middle-income countries (LMIC), was emphasized by the second edition of Disease Control Priorities (DCP) [4] and was refined further by the DCP 3 [5]. Surgical needs in LMIC have been described, mainly by using estimates and mathematical modelling [6, 7]. Population-based studies which enumerate the actual number of surgeries performed in the LMIC context are needed. This study aims to assess the surgical needs in an urban cohort of 88,273 people who are covered under an Universal Health Coverage (UHC) scheme in Mumbai, India.

Our cohort provides a near ideal setting to assess the burden of surgical diseases. It provides three main components: first, a single electronic medical records system for enumeration and documentation of surgical procedures and second, a Universal Health Coverage (UHC) system where surgeries can be performed when needed and thirdly, a setting where the number of surgeries performed are not incentivized [6]. The study also aims to test whether the LCoGS benchmark estimate of 5000 surgeries per 100,000 population is valid in an Indian urban population.

## Methods

We carried out this study in a cohort covered under, ‘Contributory Health Service Scheme’ (CHSS). This scheme is implemented for employees and their families of Department of Atomic Energy (DAE, Govt of India) and provides healthcare services at a minimum flat contribution of one percent of the basic pay of the employee. It delivers health coverage equitably, i.e. independent of economic status and individual health risks due to pre-existing illnesses [8]. The health scheme is implemented through pyramid of healthcare services consisting of 14 primary healthcare facilities, spread across the city and suburbs of Mumbai, and a central referral hospital. Referral linkages exist between these primary facilities and the central hospital, where patients are provided secondary as well as select tertiary care services like urology and basic surgical oncology. This central hospital is situated within the residential complex meant for the employees. [9] The surgery department here ‘outsources’ some specialized surgeries (neurosurgery, vascular surgery and complex surgical oncology) to tertiary care hospitals by issuing cashless vouchers to the patients. After the procedure, however, the patient returns for post-surgical care and is followed through the recovery period at the central hospital itself. This system allows for tracking and documentation of all surgeries performed on this population cohort. Our cohort of 88,273 people as a population pyramid along with the enumeration of surgeries is described in Table 1. This cohort represents an urban community and comprises employees who receive a fixed monthly salary under the central government employment, and hence, population below the poverty line is excluded. All employee families belonged to ‘upper lower’, ‘middle’ or ‘higher’ socioeconomic class as per modified Kuppuswami index [10, 11], with salaries ranging from 4,600 to 50,000 USD per year.

**Table 1:** Population and surgeries described in CHSS cohort and estimated numbers in 100,000 Indian population per year

Age Group	Study Population (CHSS)						National Population (Census of India)					
	Population			Surgeries/year, per 100,000 population			Population Distribution			Surgeries/year, per 100,000 population		
	Male	Female	Total	Male	Female	Total	Male	Female	Total	Male	Female	Total
0–4	1300	1218	2518	37	16	53	4860	4491	9351	138	61	199
5–9	1992	1754	3746	59	23	82	5496	5026	10,522	163	66	229
10–14	2177	2032	4209	52	26	78	5754	5246	11,001	137	67	205
15–19	2358	2066	4424	50	23	73	5304	4687	9991	112	52	165
20–24	2816	2581	5397	68	62	131	4773	4463	9236	116	108	224
25–29	3208	2895	6103	56	125	181	4256	4150	8407	74	179	253
30–34	2419	2451	4870	59	200	259	3702	3642	7344	91	297	388
35–39	2614	2801	5415	71	163	234	3558	3500	7058	97	204	300
40–44	2947	2953	5900	93	156	249	3112	2892	6005	99	153	252
45–49	2613	3181	5794	109	209	318	2664	2502	5166	112	164	276
50–54	2814	3755	6569	145	249	394	2142	1925	4068	110	128	238
55–59	3176	3370	6546	199	205	404	1613	1632	3245	101	100	201
60–64	2817	2912	5729	209	251	460	1550	1572	3122	115	135	251
65–69	2616	3344	5960	274	295	569	1073	1120	2193	112	99	211
70–74	2629	3129	5758	292	257	549	800	792	1592	89	65	154
75–80	2635	2520	5155	265	155	420	372	393	765	37	24	62
> 80	2456	1724	4180	134	48	183	438	498	936	24	14	38
Total	43,587	44,686	88,273	2176	2466	4642	51,467	48,531	100,000	1729	1916	3646

We retrieved data retrospectively from centralized Electronic Medical Records (EMR), common to all dispensaries and the central hospital. The surgeries which were entered in the EMR as ‘scheduled’ for booking the operation theatre time slots were enumerated. This included emergency as well as planned procedures but not bedside and emergency room procedures. The mid-term-of-study-duration community census was used to estimate age–sex-specific surgical needs. We calculated actual number of surgeries performed in each age and sex group (male/female) in our population cohort. To estimate the national needs, we age-standardized our 88,273 population for age and sex as per the national census data 2011, to estimate the standardized national surgical needs per 100,000 Indian population.

We analysed all surgeries performed over a period of 2 years starting from 01 January 2017 to 31 December 2018, which included surgeries performed at the central hospital and the outsourced surgeries. The details of the outsourced surgeries were retrieved from the cashless vouchers issued to the patients while availing tertiary care facilities at hospitals, outside of the CHSS system. Further, surgeries were categorized by specialties and as ‘essential’ or ‘non-essential’ surgeries per Disease Control Priorities, third edition [5]. The standardization of procedure nomenclature was supervised by the authors (ANG, PB, PP) to avoid duplicate entries.

## Results

A total of 9284 surgeries were performed in our population of 88,273 people over a period of 2 years; 2176 (47%) surgeries were performed on men and 2466 (53%) on women. The maximum number of surgeries (*N* = 1029, 22.1%) were performed in seventh decade (60–69 yrs.). The distribution of number of surgeries per year, categorized by age groups for both study population (CHSS) and national population, is described in Table 1. In the study population, average of 4624 surgeries were performed per year and the national estimate after standardization was 3646 surgeries per year per 100,000 Indian population. However, on adjusting for national rural and urban demographic differences, the estimated surgeries were 3640 (rural India) and 3763 (urban India) per year per 100,000 population. (Supplementary Tables 1 and 2). The national surgical estimates for men and women were 1729 and 1916 surgeries per year per 100,000 population, respectively. Within the Indian population, the maximum number of surgeries (*N* = 688, 18.8%) was estimated to be in the fourth decade (30–39 years).

**Fig.1:**
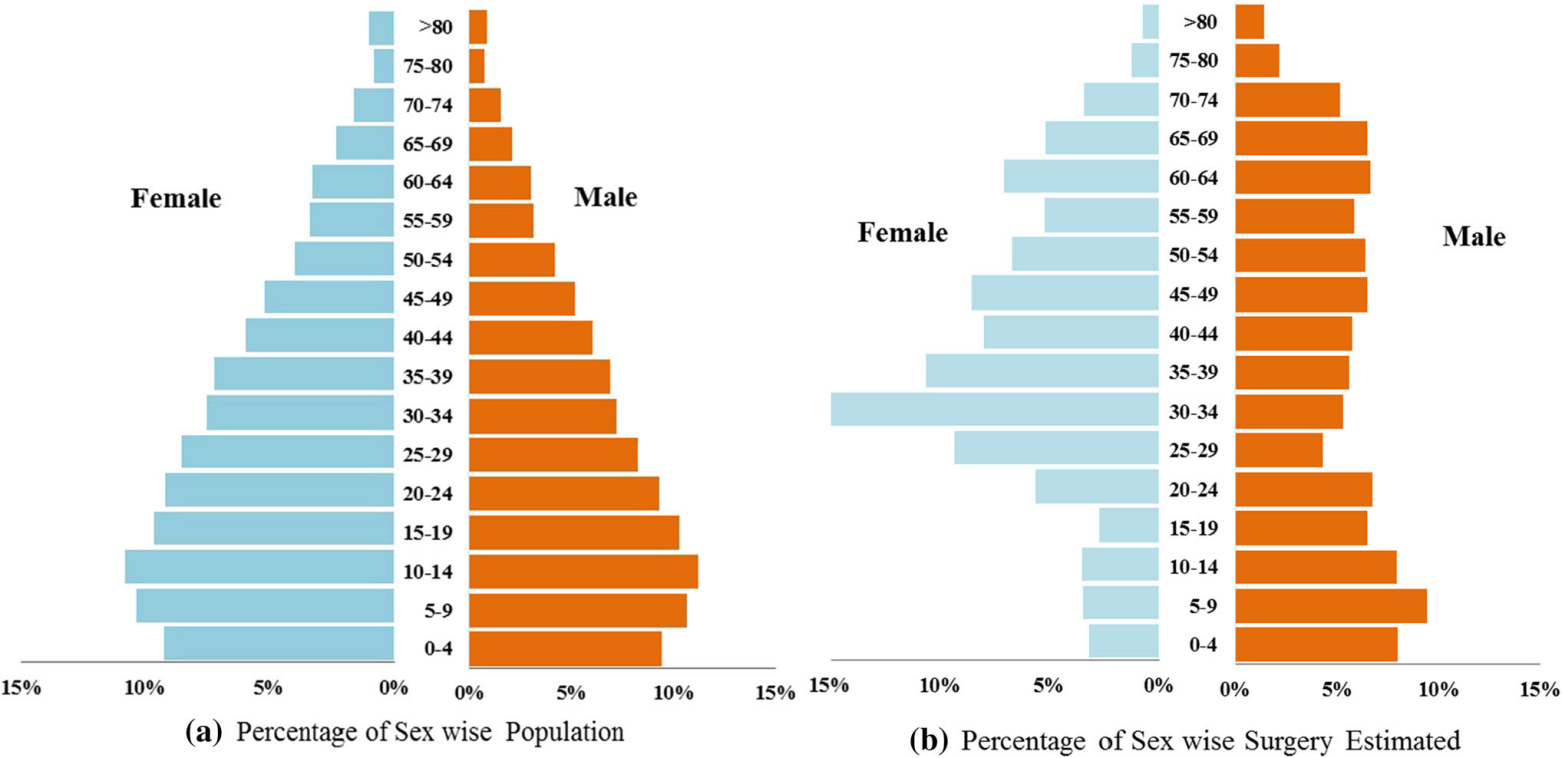
Estimated national surgical demand per year per 100,000 population; **a** Age- and sex-wise distribution of men and women and **b** Age- and sex-wise distribution of estimated surgeries amongst men and women

We estimated that Indian population (men and women together) within age groups 30–49 years will need about a third of the total number of surgeries (1216/3646, 33.3%) and 50–74 age group will need another 28% (1055/ 3646) of the total estimated surgeries (Fig. 1).

The types of surgeries performed in each specialty during the study period and the corresponding national estimates of surgeries within each specialty are described in Table 2. The commonest surgical specialties in our cohort were ophthalmology (25.3%), cataracts (22.8%) and other ophthalmic surgeries (2.5%). General surgery procedures were (18.3%) and obstetrics and gynaecology (14.1%). Amongst women, 691 gynaecological and breast surgeries accounted for one-fourth of the total surgeries performed. The specialty trend was different for the national estimates, where most surgical procedures belonged to general surgery (26.0%) followed by obstetrics and gynaecology (20.0%) and ophthalmology (13.0%).

**Table 2:** The average number of surgeries performed under different specialties classified as per DCP3

Specialties/departments	Surgeries in CHSS/year**				National Estimates of Surgeries/year*			
	Male	Female	Total	%	Male	Female	Total	%
Ophthalmology	590	585	1175	25.3	233	237	470	13
General surgery	525	326	850	18.3	614	335	949	26
Obstetrics and gynaecology	1	656	657	14.1	2	721	723	20
Musculoskeletal	291	337	628	14	289	190	479	13
Gastrointestinal system	303	201	504	11	227	133	360	10
Genitourinary system	233	68	301	6	134	39	174	5
Head, Neck-Otolaryngology	154	126	279	6	178	151	328	9
Oncology	28	51	79	2	14	27	41	1
Plastic surgery	12	63	75	2	7	40	47	1
Breast surgery	3	35	38	1	4	27	31	1
Neuro-surgery	25	9	34	1	13	4	17	0
Others	13	13	26	1	14	12	26	1
Total	2176	2466	4642		1729	1916	3646	

^*^National Population taken from 2011 Census of India. Estimates are per 100,000 populations per year [12] **Mid-year population in CHSS cohort

In our cohort, average of 2054 (44%) essential surgeries were performed per year (Table 3). When extrapolated, the national estimate was 2327 (63.8%) essential surgeries per year per 100,000 population. We performed average of 2586 non-essential surgeries in our cohort. Supplementary Table 3 describes distribution of these non-essential surgeries.

**Table 3:** Distribution of essential surgeries classified as per DCP3

	DCP3-Enlisted surgeries	Number of surgeries performed in CHSS cohort	Estimated number of surgeries per 100,000 Indian population
**General surgical**		329 (16.1%)	372 (16.1%)
	Bowel obstruction and perforation	15	17
	Colostomy	1	1
	Superficial abscess drainage	51	57
	Gall bladder surgery	44	49
	Hernia	133	151
	Hydrocele	14	16
	Male circumcision	45	51
	Relief of urinary obstruction	7	7
	Appendicectomy	20	23
Obstetric, gynaecologic, and family planning		320 (15.6%)	363 (15.6%)
	Family planning tubal ligation	88	99
	Dilatation and curettage	52	58
	Repair of obstetric fistula	2	2
	Caesarean birth	180	203
**Injury**		276 (13.4%)	313 (13.4%)
	Fracture reduction Internal fixation + external fixation	153	173
	Traumatic amputations	9	10
	Closed reduction of fractures	7	8
	Skin grafting	8	9
	Suturing of lacerations	94	106
	Tracheostomy	5	6
	Drainage of septic arthritis	1	1
**Congenital**		4 (0.2%)	4 (0.2%)
**Visual impairment**		1126 (54.8%)	1276 (54.8%)
Total		2054	2327

## Discussion

There is an acknowledged need of enumerating surgical procedures to estimate surgical needs in India.[13]. Our estimate of 3646 surgeries for Indian population is lower than the estimated need of 5000 surgeries per year per 100,000 population by the Lancet commission on Global surgery.[6] Our study estimates the total surgeries required, including surgeries categorized as essential and other surgeries assuming UHC is implemented across the country.

The national commission report from Ministry of Health, Government of India in 2005 addressed a part of surgical burden describing cataract and cancers but did not estimate the population-based needs of these surgeries [14]. Vora et al. in a recent household survey in urban population in India estimated 274 individuals needed surgery in an adult population of 10,300 which can be extrapolated to 2650 individuals needing surgery among 100,000 adults. This paper did not take into account the paediatric population and did not enumerate surgeries. The authors counted individuals needing surgeries in their population [15]. A medical college in Bangladesh, a country culturally and demographically similar to India, has reported 17,700 surgeries for 11.1 million population which would amount to approximately 160 surgeries per 100,000 Bangladeshi population [16]. This study cohort lacks a precise denominator for their population.

Estimates of surgeries for the Indian population showed that population in the fourth decade needed maximum (18%) surgeries. A study from Sierra Leone documented that people in the age group 16–33 needed highest number of surgeries (45%), suggesting a higher need in younger population [17].

The women underwent a greater number of surgeries compared to men in our cohort (1916 vs 1.729). Caesarean section rates in our cohort were 30% of all deliveries compared to the WHO suggested rates of 10%. These surgeries could partly contribute to the higher number of surgeries performed on women. A household survey in Delhi, India, carried out on 3043 residents showed that women (15.8%) underwent a greater number of surgeries compared to men (12.6%) [18]. A countrywide survey from Nepal also suggested marginally higher number of surgeries in women but did not elaborate on the reasons [19]. Contrary to this, Gyedu et al. reported higher barriers to healthcare and seeking surgeries in women compared to men in Ghana and attributed this to a lack of privacy, inability to negotiate healthcare pathways, lack of social support [20]. In our cohort, easy access and familiarity with the community hospital and primary care centres may be responsible for more women seeking surgical care than men, in comparison with Ghanaian population. The higher needs in women would need to be studied further in order to assess the number of care providers in obstetrics and gynaecology as well as general surgery.

We estimated that out of the total 3646 surgeries needed in Indian population per year, 26% would be general surgical procedures, 20% would be obstetrics and gynaecological surgeries, and 13% would be Ophthalmological surgeries. Similar studies describing proportions of specific specialty surgeries from Asia are few, but some of the pacific islands like Papua New Guinea have documented estimates of surgeries in each specialty at the country level. They have documented 783 surgeries performed per 100,000 population. The estimated percentages of general surgery, obstetrics and gynaecology and ophthalmological surgeries were 50%, 46% and 4%, respectively [21]. Similarly, higher proportion of essential gynaecology and general surgical procedures have been documented in African countries, where total volume of surgeries performed is low [22].

The estimated need of caesarean sections for Indian population was 5.5% (*N* = 203) of total surgical procedures. A community survey-based study in India carried out by Bhasin et al. reported that caesarean sections accounted for 3.2% of the total number surgeries in their urban cohort which is similar to our study [18]. Caesarean sections comprised of a third (30%) of the total surgical volume needed in very low-income countries. The proportion of caesarean sections contributed to a little over 2% of the total surgical volume in the UK [23][23]. A systematic review by Grimes et.al describing surgical volumes in sub-Saharan Africa documented huge variations (10–51%) in the proportions of caesarean sections performed [22].

We estimated 151 (4%) hernia repairs would be needed for 100,000 Indian population per year. This proportion is low compared to that observed in many African and other low-income countries. Hernia was the commonest surgery accounting for 22% of the surgeries performed in Sierra Leone. Non-availability of trained surgeons and limited training opportunities for surgeons for other complex or advanced surgical procedures in Sierra Leone could explain that essential procedures like hernias constituted larger proportions of total surgeries performed [17].

Proportion of caesarean sections and hernias of all surgeries are indicative of a mature health system. A relationship between total volumes of surgery and the fraction of caesarean sections has also been described in the literature [25, 26]. Caesarean sections and hernias together constitute a larger fraction of total surgeries in countries where low number of surgeries are observed overall [26]. The total number of surgical procedures conducted within our urban Indian cohort and estimates for India is higher compared to the other African and Asian countries, and therefore, it is expected that both caesarean sections (5%) and hernia repairs (4%) constitute a lower proportion, of all surgeries [16, 22, 23]

Ophthalmic surgeries, including cataracts (22.8%), were estimated to be 25% of the total surgeries in our cohort and 13% of the total estimated surgical need in Indian population. The cataracts accounted for 1.7% of the surgeries in Ghana and 9.9% in Sierra Leone [17, 20]. A larger proportion of the elderly in our population explains higher volume of cataract surgeries performed. Also, the cataract surgeries performed in a camp setting, which is a common setting for operating on cataracts, were not available for enumeration in these studies [17, 20].

The strength of our paper is that it assesses the volume of surgeries performed within a well-defined cohort of 88,273 people in an urban community covered under a ‘Contributory Health Service Scheme’. Availability of such a precise denominator is rather unique for low- and lower middle-income country studies. Further, the two most prominent barriers to seeking surgical healthcare (costs and travel) are nearly eliminated in this cohort, as this cohort has easy access and UHC cover. The last five years have seen the National Health Mission in India taking up non-communicable diseases as a priority issue, yet focus on surgical needs is largely lacking (except cancers). This study would provide a starting point for calculating national estimates for planners and policy makers and for future surgical enumeration studies in the country.

The authors acknowledge the limitations and assumptions in this study. The CHSS cohort differs from the Indian national population, socioeconomically and demographically. This limits the generalizability of the study partly in spite of age and sex standardization. The accessibility and affordability of the services were main assumption in the UHC service delivery. The acceptability of the services offered was also assumed to be near total. A recent study by Vora et al. in their urban cohort in Indian metropolis has documented a 50% utilization of UHC services [15], mainly due to lack of trust and lack of awareness about the UHC schemes available. We have not accounted for possibility of under-utilization of surgical services under the UHC scheme, and in that case, our figure may be an under-estimation. We did not have the data for surgeries performed at bedside in the emergency rooms, outpatient department or wards under local anaesthesia like thoracostomy tube insertion, resuscitation with surgical airway and dental surgical procedures, which is another limitation of the study. Including these missing surgeries will push our figure closer to the LCoGS estimate of 5000 surgeries.

## Conclusion

We enumerated that annually, 3646 surgeries per 100,000 population are needed to meet the surgical needs of Indian population, using an UHC population cohort. This benchmark is based on actual enumeration of surgeries against the estimated LCoGS benchmark of 5000 surgeries per 100,000 population in LMICs. Caesarean sections, hernia repairs and cataract surgeries were estimated to constitute the major proportion of the surgical needs of the urban Indian population.

## Electronic supplementary material

Below is the link to the electronic supplementary material.

Supplementary file1 (DOCX 32 kb)
