# Supplementary material for: Estimation of the National Surgical Needs in India by Enumerating the Surgical Procedures in an Urban Community Under Universal Health Coverage
Source: World J Surg. 2020 Sep 24;45(1):33–40. doi: 10.1007/s00268-020-05794-7 (PMC7752785; doi:10.1007/s00268-020-05794-7)
Supplement: Supplementary file 1 — Supplementary file1 (DOCX 32 kb) [file 268_2020_5794_MOESM1_ESM.docx]

Supplementary table 1: Age and sex standardized surgeries estimated for 100,000 urban Indian population.

|  | Study Population – CHSS | | | | | | Urban Indian Scenario | | | | | |
| --- | --- | --- | --- | --- | --- | --- | --- | --- | --- | --- | --- | --- |
|  | Population | | | Procedures | | | 100,000 Population Breakup | | | Procedures/Year/100,000 | | |
| Age | Male | Female | Total | Male | Female | Total | Male | Female | Total | Male | Female | Total |
| 0-4 | 1300 | 1218 | 2518 | 37 | 17 | 54 | 3788 | 4153 | 7941 | 108 | 56 | 169 |
| 5-9 | 1992 | 1754 | 3746 | 59 | 23 | 82 | 4166 | 4653 | 8820 | 123 | 61 | 193 |
| 10-14 | 2177 | 2032 | 4209 | 52 | 26 | 78 | 4520 | 5041 | 9561 | 108 | 65 | 177 |
| 15-19 | 2358 | 2066 | 4424 | 50 | 23 | 73 | 4583 | 5169 | 9753 | 97 | 58 | 161 |
| 20-24 | 2816 | 2581 | 5397 | 69 | 63 | 131 | 4831 | 5178 | 10010 | 118 | 125 | 243 |
| 25-29 | 3208 | 2895 | 6103 | 56 | 125 | 181 | 4627 | 4785 | 9412 | 81 | 207 | 279 |
| 30-34 | 2419 | 2451 | 4870 | 60 | 200 | 260 | 3983 | 4188 | 8171 | 98 | 342 | 435 |
| 35-39 | 2614 | 2801 | 5415 | 71 | 163 | 234 | 3804 | 3939 | 7743 | 103 | 229 | 335 |
| 40-44 | 2947 | 2953 | 5900 | 94 | 156 | 250 | 3163 | 3456 | 6619 | 100 | 183 | 280 |
| 45-49 | 2613 | 3181 | 5794 | 110 | 209 | 319 | 2758 | 3002 | 5760 | 116 | 197 | 317 |
| 50-54 | 2814 | 3755 | 6569 | 145 | 250 | 395 | 2126 | 2411 | 4537 | 110 | 160 | 272 |
| 55-59 | 3176 | 3370 | 6546 | 199 | 206 | 405 | 1695 | 1843 | 3538 | 106 | 112 | 219 |
| 60-64 | 2817 | 2912 | 5729 | 210 | 251 | 461 | 1492 | 1537 | 3028 | 111 | 132 | 243 |
| 65-69 | 2616 | 3344 | 5960 | 274 | 296 | 570 | 1013 | 995 | 2007 | 106 | 88 | 192 |
| 70-74 | 2629 | 3129 | 5758 | 293 | 257 | 550 | 726 | 712 | 1438 | 81 | 58 | 137 |
| 75-80 | 2635 | 2520 | 5155 | 266 | 155 | 421 | 538 | 469 | 1007 | 54 | 29 | 82 |
| >80 | 2456 | 1724 | 4180 | 135 | 49 | 183 | 354 | 302 | 655 | 19 | 8 | 29 |
| Total | 43587 | 44686 | 88273 | 2176 | 2466 | 4642 | 48167 | 51833 | 100000 | 1639 | 2111 | 3763 |

Supplementary table 2: Age and sex standardized surgeries estimated for 100,000 rural Indian population.

|  | Study Population – CHSS | | | | | | Rural Indian Scenario | | | | | |
| --- | --- | --- | --- | --- | --- | --- | --- | --- | --- | --- | --- | --- |
|  | Population | | | Procedures | | | 100,000 Population Breakup | | | Procedures/Year/100,000 | | |
| Age | Male | Female | Total | Male | Female | Total | Male | Female | Total | Male | Female | Total |
| 0-4 | 1300 | 1218 | 2518 | 37 | 17 | 54 | 5180 | 4808 | 9988 | 147 | 65 | 212 |
| 5-9 | 1992 | 1754 | 3746 | 59 | 23 | 82 | 5877 | 5414 | 11291 | 174 | 71 | 247 |
| 10-14 | 2177 | 2032 | 4209 | 52 | 26 | 78 | 6077 | 5575 | 11651 | 145 | 71 | 216 |
| 15-19 | 2358 | 2066 | 4424 | 50 | 23 | 73 | 5365 | 4734 | 10099 | 114 | 53 | 167 |
| 20-24 | 2816 | 2581 | 5397 | 69 | 63 | 131 | 4590 | 4296 | 8887 | 112 | 104 | 216 |
| 25-29 | 3208 | 2895 | 6103 | 56 | 125 | 181 | 4017 | 3935 | 7952 | 70 | 170 | 236 |
| 30-34 | 2419 | 2451 | 4870 | 60 | 200 | 260 | 3483 | 3488 | 6970 | 86 | 285 | 371 |
| 35-39 | 2614 | 2801 | 5415 | 71 | 163 | 234 | 3385 | 3363 | 6748 | 92 | 196 | 292 |
| 40-44 | 2947 | 2953 | 5900 | 94 | 156 | 250 | 2957 | 2770 | 5727 | 94 | 146 | 242 |
| 45-49 | 2613 | 3181 | 5794 | 110 | 209 | 319 | 2511 | 2386 | 4897 | 105 | 157 | 269 |
| 50-54 | 2814 | 3755 | 6569 | 145 | 250 | 395 | 2021 | 1835 | 3855 | 104 | 122 | 232 |
| 55-59 | 3176 | 3370 | 6546 | 199 | 206 | 405 | 1509 | 1604 | 3113 | 95 | 98 | 192 |
| 60-64 | 2817 | 2912 | 5729 | 210 | 251 | 461 | 1556 | 1608 | 3164 | 116 | 139 | 254 |
| 65-69 | 2616 | 3344 | 5960 | 274 | 296 | 570 | 1108 | 1168 | 2277 | 116 | 103 | 218 |
| 70-74 | 2629 | 3129 | 5758 | 293 | 257 | 550 | 840 | 822 | 1662 | 93 | 68 | 159 |
| 75-80 | 2635 | 2520 | 5155 | 266 | 155 | 421 | 536 | 575 | 1111 | 54 | 35 | 91 |
| >80 | 2456 | 1724 | 4180 | 135 | 49 | 183 | 292 | 315 | 607 | 16 | 9 | 27 |
| Total | 43587 | 44686 | 88273 | 2176 | 2466 | 4642 | 51303 | 48697 | 100000 | 1733 | 1891 | 3640 |

Supplementary table 3: List of non- essential surgeries in CHSS cohort

| Specialty or Group | Types of different procedures included | Year 2017 | Year 2018 | Average Per Year |
| --- | --- | --- | --- | --- |
| CVTS | 1 | 15 | 5 | 20 |
| ENT | 49 | 295 | 274 | 569 |
| Eyes | 15 | 40 | 39 | 79 |
| General Surgery | 75 | 1089 | 995 | 2084 |
| Obstetrics and Gynecology | 41 | 319 | 347 | 666 |
| Oncology | 18 | 115 | 121 | 236 |
| Orthopedics | 41 | 494 | 490 | 984 |
| Urology | 18 | 303 | 231 | 534 |
| Total |  | 2670 | 2502 | 2586 |
